# Risk assessment for injuries resulting from positioning and incidence of neuropathy and skin lesions

**DOI:** 10.1590/0034-7167-2025-0126

**Published:** 2026-09-28

**Authors:** Renata Elizabete Pagotti da Fonseca, Thamires Carraro Gatto, Carina Aparecida Marosti Dessotte

**Affiliations:** I Universidade de São Paulo. Ribeirão Preto, São Paulo, Brazil; II Universidade Federal de São Carlos. São Carlos, São Paulo, Brazil

**Keywords:** Pressure Ulcer, Small Fiber Neuropathy, Patient Positioning, Perioperative Nursing, Patient Safety., Úlcera por Presión, Neuropatía de Fibras Pequeñas, Posicionamiento del Paciente, Enfermería Perioperatoria, Seguridad del Paciente.

## Abstract

**Objectives:**

to assess the risk of developing injuries resulting from surgical positioning in adult patients undergoing elective surgery and to identify the incidence of neurological complications and skin lesions in the perioperative period.

**Methods:**

an observational, longitudinal, prospective, and quantitative study conducted in a medium-sized hospital in the countryside of São Paulo, Brazil.

**Results:**

the study sample was predominantly female (57%), with a median age of 55 years. Of these, 72.8% had pre-existing conditions, with overweight (41.8%) or obesity (34.8%). The Risk Assessment Scale for the Development of Injuries Resulting from Surgical Positioning score was low risk (75.9%), with an incidence of 6.4% of peripheral neuropathy and 0.8% of pressure injury (stage 1/sacral region).

**Conclusions:**

using risk assessment scales for the development of injuries helps to systematize nursing care. Studies on the incidence of injuries (skin and peripheral neuropathy) are essential for situational diagnosis and subsequent creation of strategies to reduce risks.

## RESUMEN

**Objetivos:**

evaluar el riesgo de desarrollar lesiones derivadas del posicionamiento quirúrgico en pacientes adultos sometidos a cirugía electiva e identificar la incidencia de complicaciones neurológicas y lesiones cutáneas en el periodo perioperatorio.

**Métodos:**

estudio observacional, longitudinal, prospectivo y cuantitativo, realizado en hospital de tamaño mediano en el interior de São Paulo, Brasil.

**Resultados:**

la muestra del estudio fue predominantemente femenina (57%), con una edad media de 55 años. De ellas, el 72,8% presentaba afecciones preexistentes, con sobrepeso (41,8%) u obesidad (34,8%). La puntuación en la Escala de Evaluación de Riesgos para el Desarrollo de Lesiones por Posicionamiento Quirúrgico fue de bajo riesgo (75,9%), con una incidencia del 6,4% de neuropatía periférica y del 0,8% de úlceras por presión (etapa 1/región sacra).

**Conclusiones:**

el uso de escalas de evaluación de riesgos para el desarrollo de lesiones ayuda a sistematizar la atención de enfermería. Los estudios sobre la incidencia de lesiones (cutáneas y neuropatía periférica) son esenciales para el diagnóstico situacional y la posterior creación de estrategias para reducir los riesgos.

## INTRODUCTION

In healthcare, quality and patient safety have special significance due to the characteristics of the work process^(1)^. In the operating room, it is essential to focus on perioperative complications in order to adopt strategies that minimize risks and promote excellence in care.

This study focuses on complications arising from surgical positioning. Several risk factors, such as age, weight, skin condition, nutritional status, comorbidities (especially involving the circulatory, respiratory, and neurological systems), immunosuppression, and physical limitations, must be investigated. Intraoperative factors such as type of anesthesia, duration of surgery, and surgical position should also be assessed^(2,3)^. Special attention should be given to elderly, obese, and pregnant patients^(2,4)^.

Scales have been developed and validated to assess the risk of injuries related to surgical positioning. This study used the Risk Assessment Scale for the Development of Injuries Resulting from Surgical Positioning (ELPO - *Escala de Avaliação de Risco para o Desenvolvimento de Lesões Decorrentes do Posicionamento Cirúrgico*), developed in Brazil, which includes seven items: type of positioning on the operating table; duration of surgery; type of anesthesia; support surface; limb positioning; comorbidities; and patient age^(5)^.

Although these complications may affect several systems, such as circulatory, respiratory, osteoarticular, muscular, neurological, and integumentary systems^(6)^, this study investigated integumentary injuries and peripheral neuropathies.

Patients are at high risk of developing pressure injuries (PIs) during surgical or invasive procedures due to prolonged immobility in the perioperative period^(3)^. Physical forces such as pressure, shear, microclimate, and friction, used to maintain proper surgical positioning, may damage the skin and underlying tissues. Additionally, operating room conditions such as humidity and temperature increase skin vulnerability^(3)^. Anesthetized patients may not feel pain or discomfort during procedures^(2)^.

PIs resulting from surgical positioning are preventable adverse events, with incidence ranging from 4% to 45%^(7)^. This variation may be due to difficulty in associating these injuries with intraoperative positioning, especially because of delayed manifestation, which may occur up to five days later and may be confused with other causes unrelated to surgery^(8)^.

During the perioperative period, patients may also develop iatrogenic peripheral neuropathy, defined as injury to nerves responsible for transmitting signals to and from the central nervous system and spinal cord. The main causes include peripheral nerve blocks and improper patient positioning^(9)^. Between 1990 and 2013, analysis of the American Society of Anesthesiologists (ASA) claims database showed that the incidence of neurological injury in patients under general anesthesia was 12%^(10)^.

Peripheral nerves may be injured during positioning, compromising sensory and motor functions. The main mechanisms include compression, ischemia, and stretching^(11)^. Risk factors include patient characteristics and anesthetic and surgical factors^(3,4)^. Perioperative peripheral neuropathy is assessed by postoperative signs and symptoms such as paresthesia, muscle weakness, tingling, or pain in the extremities^(12)^.

These complications impact healthcare costs and patients’ and families’ quality of life^(13)^. Therefore, it is essential to assess the risk of injuries related to surgical positioning and investigate neurological and skin complications in elective surgeries. Situational diagnosis helps professionals identify risks and supports decision-making. Furthermore, there is a gap in studies on peripheral neuropathy in perioperative nursing care.

Thus, the following research questions were formulated: what is the risk classification for the development of injuries related to surgical positioning in adult patients undergoing elective surgeries in a university hospital in the countryside of São Paulo? What is the incidence of neurological injuries related to surgical positioning (iatrogenic peripheral neuropathy) and skin injuries (PI) in these patients?

## OBJECTIVES

To assess the risk for the development of injuries related to surgical positioning in adult patients undergoing elective surgeries and to identify the incidence of such injuries, including iatrogenic peripheral neuropathy and skin injuries (PI), in adult patients undergoing elective surgeries in a university hospital in the countryside of São Paulo.

## METHODS

### Ethical aspects

This study follows the Brazilian standards established by Resolutions 466/2012^(14)^ and 510/2016^(15)^ of the Brazilian National Health Council, which regulate research involving human beings. The project was submitted to the Research Ethics Committee and received a favorable opinion. The participation of those involved in the research occurred through the signing of the Informed Consent Form.

### Study design, period, and setting

This is an observational, longitudinal, prospective study conducted in a medium-complexity university hospital in the countryside of São Paulo, following *STrengthening the Reporting of OBservational studies in Epidemiology* guidelines^(16)^. The hospital has five operating rooms and performs approximately 150 surgeries per month.

Data collection took place from July to December 2024 and was conducted as follows: sociodemographic and clinical data, as well as preoperative data, were collected in the operating room’s consultation room. During the intraoperative period, data collection occurred from the patients’ entry into the operating room until their transfer to the post-anesthesia recovery room. During this period, data related to the anesthetic-surgical procedure were collected, including the application of ELPO. In the postoperative period, to identify possible neuropathic and integumentary lesions, patients were assessed during transfer from the operating table to the stretcher, in the immediate postoperative period, in the post-anesthesia recovery room, and daily until the day of hospital discharge.

### Sample, inclusion and exclusion criteria

A consecutive, non-probabilistic sample consisted of patients of both sexes, over 18 years of age, who underwent elective surgical procedures in the specialties of general surgery, head and neck surgery, gynecology, mastology, orthopedics, ophthalmology, proctology, urology, and vascular surgery. To address the second objective, patients with pre-existing peripheral neuropathy and PI prior to entering the operating room were excluded.

### Study protocol

To collect sociodemographic and clinical data, as well as pre-, intra-, and postoperative periods, an instrument adapted from Lopes *et al*. was used, covering the following variables^(5)^: date of birth; date of surgery; name of surgery; biological sex; weight; height; physical status classification according to the ASA; and presence of pre-existing diseases (diabetes mellitus, previous vascular disease, previous neuropathy, deep vein thrombosis, hypertension (HT), previous PI). All variables were collected from patients’ medical record. Age was calculated by subtracting the date of birth from the date of the interview. Body Mass Index (BMI) was calculated using the collected weight and height. For nutritional classification in adults, the BMI parameter recommended by the World Health Organization was considered^(17)^.

The variable “presence of previous peripheral neuropathy” was collected from patients’ medical record, considering preoperative assessment, and was classified as “presence of previous peripheral neuropathy: yes or no”.

To obtain the variable “previous PI”, the researcher performed patients’ preoperative assessment, through inspection of the entire body surface, and it was classified as “presence of previous PI: yes or no”.

The following preoperative variables were also collected: presence of pain (yes or no); location; pain assessment using a numerical scale; presence of PI (yes or no); and previous history of PI (yes, no, location). In the intraoperative period, the variables “time of onset of anesthesia and surgery” and “time of completion of anesthesia and surgery” were collected.

During the intraoperative period, ELPO^(5)^ was applied, consisting of seven items: type of surgical position; surgery time; type of anesthesia; support surface; limb position; comorbidities; and patient age. Each item has five sub-items, with scores ranging from 1 to 5. The final score obtained was the scale, which can range from 7 to 35 points. The higher the score, the greater the risk of developing PIs resulting from surgical positioning. A score of 20 is considered the cut-off point to differentiate patients as follows: scores between 7 and 19 points are considered lower risk; and scores between 20 and 35 points are considered higher risk for developing PIs resulting from surgical positioning^(5)^.

In the postoperative period, the following data were collected: presence of pain; paresthesia; muscle weakness; and tingling in a location other than the surgical site. Considering the concept of peripheral neuropathy adopted in the study, patients were classified as having “yes” to the presence of nerve injury - peripheral neuropathy - if they presented at least one of these signs at any time during data collection in the postoperative period.

Furthermore, the skin was assessed for the presence of marks left by sheets or equipment accessories, blanchable erythema, and skin lesions. To identify PIs, the entire extent of the patients’ skin was assessed by inspection and palpation at all assessment periods. The National Pressure Injury Advisory Panel criteria were used to classify the stage of PIs^(18,19)^. To make the study replicable, the step-by-step process is reproduced in Figure 1.

**Figure 1:**
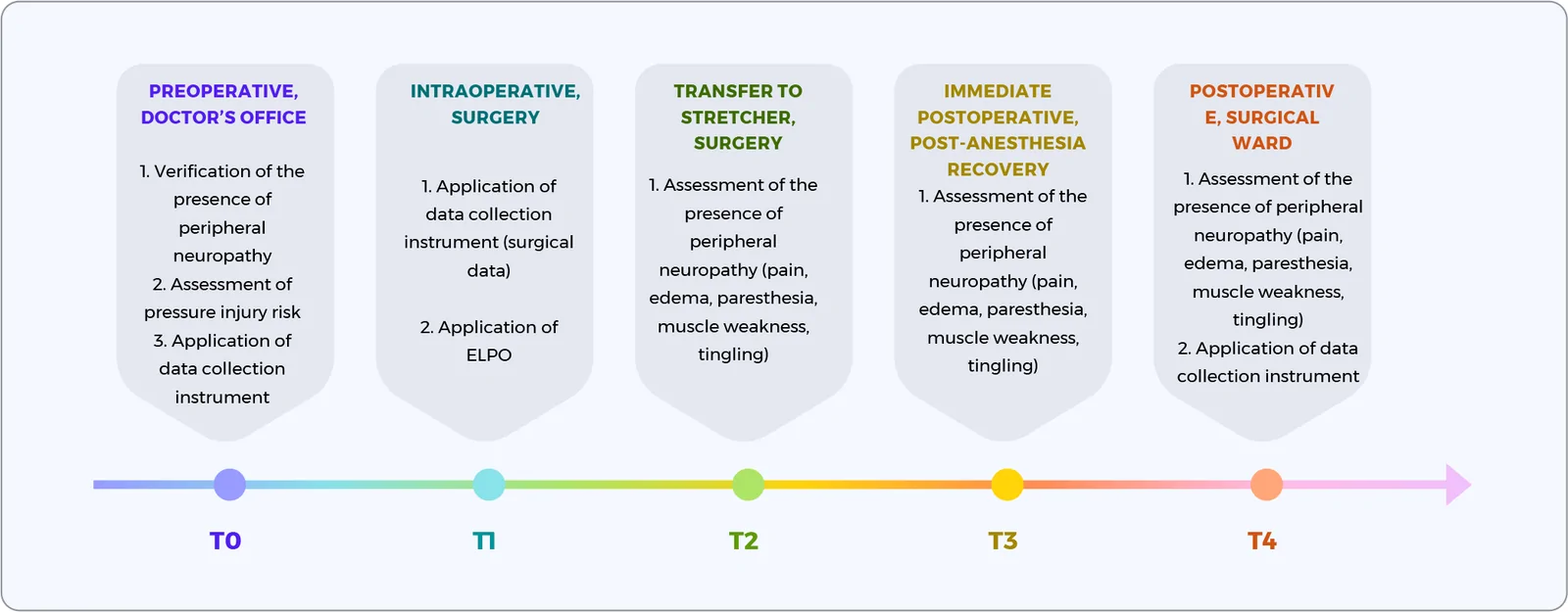
Timeline of data collection, 2025

## RESULTS

### Assessment of the risk of injury resulting from surgical positioning

To address the first objective, which is to assess the risk of developing lesions resulting from surgical positioning, the sample consisted of 158 patients, the majority of whom were female (n=90, 57%). Ages ranged from 20 to 82 years, with a median of 55 years. Most patients were classified as ASA II (n=111, 70.3%), followed by ASA I (n=40, 25.3%) and ASA III (n=7, 4.4%). In the nutritional assessment according to the BMI of the interviewed patients, the majority of patients interviewed were overweight (n=66, 41.8%), followed by obesity (n=55, 34.8%) and, finally, normal weight (n=37, 23.4%).

In relation to pre-existing conditions, 72.8% of the sample (n=115) reported having some pre-existing condition before undergoing the surgical procedure. There was a prevalence of hypertensive patients (n=63, 39.9%) or with another vascular disease (n=48, 30.4%), followed by diabetics (n=34, 21.5%) and those with peripheral neuropathy (n=33, 20.9%). Moreover, a small sample (n=25, 15.8%) reported being smokers.

In the variable “occupation of research participants”, the highest frequency was for activities performed at home (n=30, 18.9%), followed by auxiliary activities (n=23, 14.5%) and retirees (n=20, 12.6%), as shown in Table 1.

**Table 1:** Frequency of occupations of research participants, São Carlos, São Paulo, Brazil

Occupation	Frequency
Retired	20 (12.6%)
Service assistant (assembly, production, cleaning)	23 (14.5%)
Administrative assistant (secretary)	4 (2.5%)
Carpenter/gardener/metalworker/joiner	8 (5.0%)
Shopkeeper	15 (9.5%)
Cook/baker/seamstress	12 (7.6%)
Unemployed	1 (0.6%)
Homemaker	30 (18.9%)
Student	4 (2.5%)
Driver	5 (3.2%)
Painter/electrician/bricklayer	9 (5.7%)
Agricultural producer	3 (2.0%)
Teacher	3 (2.0%)
Healthcare professional	12 (7.6%)
Security guard/janitor/caretaker	6 (3.8%)
Public servant	3 (2.0%)
**TOTAL**	**158 (100%)**

During the data collection period, the specialty that performed the most procedures was general surgery, including video-assisted cholecystectomies, lesion excisions, and hernioplasties (n=52, 32.9%), followed by gynecological surgeries, with an emphasis on colpoperineoplasties, endometrioma excisions, hysterectomies, and hysteroscopies (n=24, 15.2%). Other specialties included vascular surgery (n=22, 13.9%), orthopedics (n=18, 11.4%), ophthalmology (n=17, 10.7%), urology (n=12, 7.6%), proctology (n=5, 3.2%), and mastology (n=5, 3.2%). It is worth noting, to understand the size of the institution, that the ophthalmological surgeries were exclusively for pterygium; the orthopedic surgeries, for carpal tunnel syndrome; and the head and neck surgeries, for parathyroidectomy and thyroidectomy. Mastology surgeons, for quadrantectomy; and vascular surgeons, for surgical treatment of varicose veins.

The risk analysis of these research participants showed that the majority presented a low-risk score (n=120, 75.9%). Figure 2 highlights the scale score according to the absolute number of patients.

**Figure 2:**
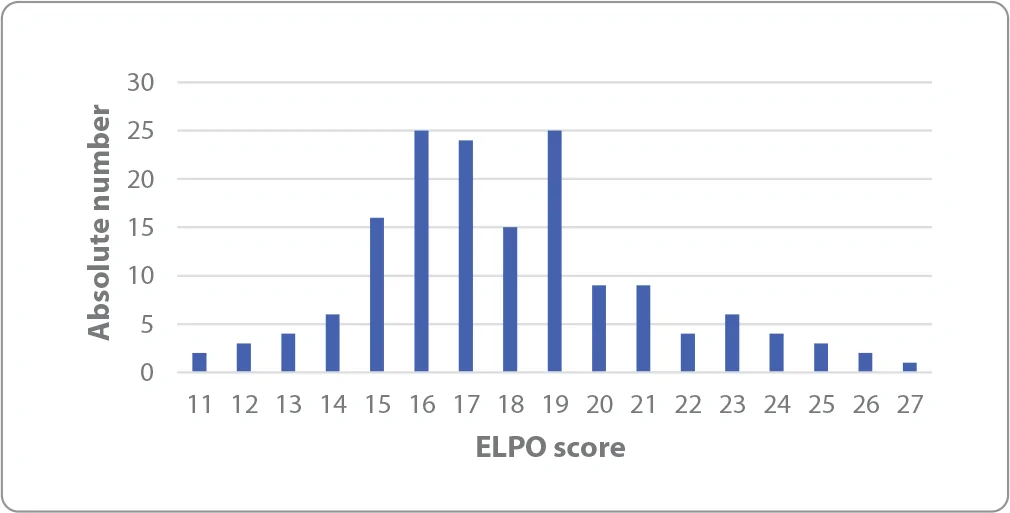
Scores of research participants according to the Risk Assessment Scale for the Development of Injuries Resulting from Surgical Positioning (*Escala de Avaliação de Risco para o Desenvolvimento de Lesões Decorrentes do Posicionamento Cirúrgico*), 2025

Most procedures were performed under spinal anesthesia (n=75, 47.5%). The most prevalent surgical position was supine (n=102, 64.6%), and most surgeries lasted up to one hour (n=79, 50%) or, at most, two hours (n=60, 38%), indicating that the hospital handles minor and medium-sized surgeries. The operating table mattress and cotton cushions were used as support surfaces, and most upper limbs were positioned with an opening of less than 90º (n=132, 83.5%), as specified in Table 2.

**Table 2:** Presentation of the risk assessment for injury development according to frequency in the studied sample, São Carlos, São Paulo, Brazil

Items assessed on the scale		Frequency
**Type of surgical position**	Supine	102 (64.6%)
	Side	6 (3.8%)
	Trendelenburg	17 (10.8%)
	Prone	8 (5.1%)
	Lithotomy	25 (15.8%)
**Surgery time**	Up to one hour	79 (50%)
	Over one hour up to two hours	60 (38%)
	Over two hours up to four hours	18 (11.4%)
	Over four hours up to six hours	1 (0.6%)
	Over six hours	0 (0%)
**Type of anesthesia**	Local	37 (23.4%)
	Sedation	13 (8.2%)
	Regional	75 (47.5%)
	General	30 (19%)
	General and regional	3 (1.9%)
**Support surface**	Surgical table mattress (conventional foam)	158 (100%)
**Limp position**	Lower limb opening less than 90º	132 (83.5%)
	Knee elevation greater than 90º	26 (16.5)
**Comorbidities**	No comorbidities	38 (24.1%)
	Vascular disease	29 (18.4%)
	Diabetes Mellitus	10 (6.3%)
	Malnutrition or obesity	48 (30.4%)
	Peripheral neuropathy	33 (20.9%)
**Comorbidities**	Age between 18 and 39	36 (22.8%)
	Age between 40 and 59	71 (44.9%)
	Age between 60 and 69	37 (23.4%)
	Age between 70 and 79	12 (7.6%)
	Age over 80	2 (1.3%)

It is important to highlight the concern regarding initiating longer surgical procedures at this institution without implementing strategies to prevent these complications, as the increased complexity and surgical time would further elevate the risk in this sample, which already presents other associated diseases, in addition to overweight and obesity.

### Incidence of neurological complications and skin lesions

To identify the incidence of neurological complications and skin lesions in the perioperative period, 33 patients from the initial sample were excluded at this stage because they had pre-existing peripheral neuropathy. It was important to include them in the first phase of the study because patients with peripheral neuropathy have a higher score on the risk scale. If they had been excluded at this first stage, the risk of developing lesions would have been skewed.

Thus, in the second phase, 125 patients participated, 65 of whom were female (52.4%). To identify the incidence of peripheral neuropathy, patients who already had the diagnosis and/or who presented to the healthcare service with pain unrelated to the surgical site on the day of surgery were excluded. No research participant presented muscle weakness or tingling in the extremities, however, some patients presented pain and paresthesia after the intervention. Thus, the incidence of peripheral neuropathy in this study was 6.4% (n=8), with seven patients presenting pain, and one patient, paresthesia. Of these patients, two presented pain in the right shoulder, three in the right upper limb (RUL), one in the right shoulder and RUL, and one patient presented pain in the left thigh. It is important to emphasize that the patient who reported pain in the left thigh had undergone a surgical procedure in the lithotomy position.

Regarding skin lesions resulting from surgical positioning, skin inspection was performed by observing three signs: marks from bed sheets or equipment accessories on the skin; blanchable erythema; and PI. It was found that 73.6% of patients, at the time of transfer from the operating table to the stretcher, had skin marks from bed sheets or equipment accessories (sphygmomanometer, pulse oximeter, scalpel blade) and 31.2% presented blanchable erythema in some part of the body at the same time of inspection. In the immediate postoperative period, 29.6% of the sample still had sheet marks (n=37), and 10.4% still presented with blanchable erythema (n=13). Only one patient (0.8%) presented with stage 1 PIs in the sacral region. This patient was a 51-year-old overweight adult with HT, who had undergone spinal anesthesia and a urological procedure, and had a high-risk ELPO score.

As for the locations where these marks and erythema appeared, the dorsal region stands out, with marks from sheets and equipment accessories, and the sacral region, with the presence of whitish erythema immediately after the end of surgery, at the moment of transferring the patient from the operating table to the stretcher, as shown in Figure 3.

**Figure 3:**
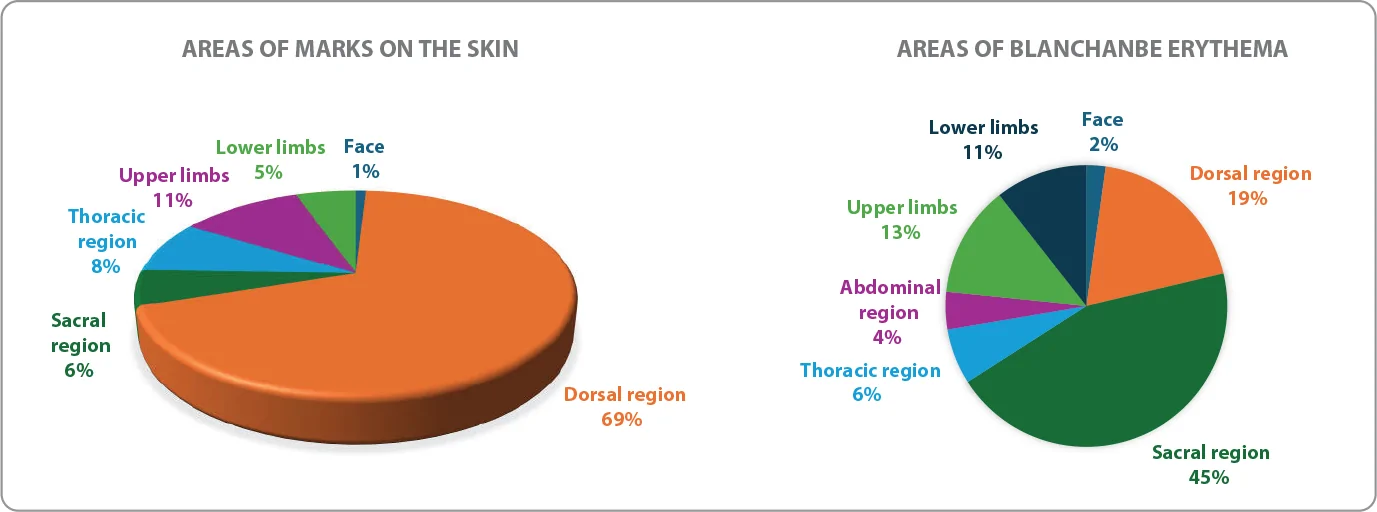
Areas with skin tags and blanching erythema in the studied sample, 2025

In this studied sample, the length of hospital stay varied, with same-day discharge (n=71, 56.8%), one-day hospitalization (n=50, 40%), two days (n=2, 1.6%), three days (n=1, 0.8%), and four days (n=1, 0.8%). It is worth noting that patients who presented with PI were the ones who stayed hospitalized for the longest time (four days).

## DISCUSSION

In the present study, the majority of participants were female (57%) and had comorbidities (72.8%), which are known to increase the risk of developing PIs resulting from surgical positioning^(6)^. The most frequent diseases were HT (39.9%) and other vascular diseases (30.4%).

HT affects blood circulation, compromising the sensory and oxygenation capabilities of organs and tissues, becoming a risk factor for PI^(20,21)^. Patients with diabetes mellitus (21.5% of the sample) also have a higher risk of PI due to vasculopathy, which decreases blood flow and compromises tissue perfusion^(20,22)^.

Comorbidities such as vascular and respiratory diseases, neuropathies, and malnutrition increase the fragility of the body’s systems. The more severe these conditions, especially those affecting tissue perfusion and oxygenation, the greater the risk of surgical positioning injuries^(6)^. Assessing the risks of perioperative complications is essential for patients, families, and the multidisciplinary team to choose the best care during the perioperative period.

One concerning result was the high prevalence of overweight (41.8%) and obesity (34.8%). Obesity is an important consideration in the perioperative setting, as its incidence is increasing, and obese patients have decreased respiratory reserve and an increased risk of complications in surgical positioning^(3)^. The perioperative nursing team should offer the best possible care to this population and adopt strategies to ensure optimal positioning. A study highlighted the need to assess bioimpedance as a risk factor for the development of PIs, considering the pressure distribution in regions prone to contact with supporting surfaces^(23)^.

Another risk factor considered was age, with 32.3% of the sample being elderly. Elderly patients present physiological changes, loss of skin elasticity, and impaired tissue nutrition and oxygenation^(20,22-24)^, making them more susceptible to complications from surgical positioning.

In the risk score assessment, low risk prevailed (75.9%). Despite the presence of risk factors such as overweight, obesity, and comorbidities, surgeries were generally short: 50% lasted less than one hour, and 38% up to two hours. This highlights the importance of training programs to assess risks, apply appropriate procedures, transfer care to other units, and report adverse events, ensuring patient safety^(2)^. With increasing surgical complexity, the risk may increase, highlighting the need for new strategies, the use of appropriate support surfaces, prophylactic materials^(2)^, and education of patients and staff while the risk is low. In the era of innovation and new technologies in healthcare, nursing must be equipped with new knowledge to provide quality care and ensure patient safety in the perioperative period.

This study identified the supine position as the most prevalent, as did other studies, which also indicate it as the most commonly used position in most surgical procedures, in addition to being the position of choice for anesthesia induction^(6,25-26)^.

The distinguishing feature of this study was that, in addition to assessing the incidence of skin lesions, it focused on peripheral neuropathies. There are few studies in perioperative nursing that assess the incidence of peripheral neuropathies in the perioperative period. A Brazilian study in the field of anesthesiology reported cases of peripheral neuropathy after bariatric surgery^(27)^.

The incidence of peripheral neuropathy in this study was 6.4%. This result highlights the need for continuous monitoring and appropriate management strategies for patients with peripheral neuropathy after surgery, aiming to reduce the impact on quality of life and promote rehabilitation. Perioperative nursing should conduct further studies on this topic, especially because peripheral nerve injuries can evolve into persistent, severe, and treatment-refractory neuropathic pain (NP), affecting patients’ quality of life^(28)^.

A review showed that chronic postoperative pain is experienced by 10% to 50% of individuals after classic operations, and can be severe in about 5% to 10% of these patients, with 20% seeking a pain clinic due to chronic postoperative pain. The incidence of NP varies between 6% and 68%, depending on the type of surgery^(29)^.

This study addresses neuropathy as a complication after hip arthroplasty, affecting up to 3% of patients and resulting in NP and muscle weakness. The study emphasizes the importance of early and multidisciplinary rehabilitation to minimize neuropathic damage related to surgical manipulation^(30)^.

The incidence of PIs was 0.8% (stage 1 lesion in the sacral region). However, 73.6% of patients presented with skin marks from sheets or accessories during transfer from the operating table to the stretcher, and 31.2% had erythema on some part of their body at that time. A similar study found the same rate of PIs in the sacral region^(24)^.

To avoid skin marks, perioperative nurses should ensure that the bed surface is smooth and wrinkle-free before positioning patients^(2)^. A 1989 study already indicated that sacral pressure increases with additional layers, such as folded sheets and incontinence pads, in patients in the supine position^(31)^.

Correct positioning is essential for surgery, ensuring good exposure of the surgical site, but it must maintain body anatomy and be physiologically tolerated. This involves the surgeon’s preference, the anesthesiologist’s needs, and the patient’s pre-existing conditions. The combination of these factors can increase the risk of postoperative complications, whether cutaneous or neuropathic^(4-6)^.

A Brazilian study found PIs in 5.47% of 128 patients followed up during the intraand postoperative periods in a large hospital^(32)^. Another study, with 52 patients, found that 7.7% developed PIs resulting from the surgical procedure. In this study, the majority of patients (65.4%) also had a low risk of positioning injury and used a conventional foam mattress, which is similar to the present study^(25)^.

Studies on the incidence of complications are essential, as their results can guide strategies to reduce risks. Research with 944 patients at high risk for positioning injuries showed a reduction in the incidence of these injuries (from 4.8% to 1.6%) after the implementation of preventive measures, such as patient education, application of protective dressings, control of skin moisture, and use of supportive surfaces^(8,33)^.

The operating room nursing team should routinely assess patients for PIs after surgery and ensure appropriate follow-up, including transferring care based on these signs. They should assess the integumentary system for erythema that disappears with pressure, changes in skin temperature, texture, or sensation of pain^(34)^, as well as investigate pain, paresthesia, muscle weakness, and tingling in areas outside the surgical site.

However, the nursing team faces challenges in promoting humanized care in the operating room. A systematic review highlighted challenges such as work overload, pressure for efficiency, the impact of technology on humanization, education and training in humanization and correct patient positioning, as well as cultural and organizational barriers^(8)^. Perioperative nursing must be prepared to overcome these challenges, always prioritizing patient safety.

The nursing team already has guidelines for managing the risk of surgical positioning injuries^(2,4,12,19,34)^, with guidelines to prevent complications in patients undergoing surgical and invasive procedures. It is essential to strengthen healthcare services and train perioperative professionals to implement complication prevention programs, perform preoperative risk assessments^(20)^, use support surfaces^(8)^ and prophylactic materials, maintain effective communication during the transfer of care, and establish policies and procedures^(22)^ that ensure quality of care and the education of patients and staff.

However, there are still challenges in the pursuit of patient safety. Some institutions lack specialized devices for intraoperative pressure relief, resorting to adaptations with materials such as foam, cotton and sheets to reduce tissue interface pressure^(25)^.

This work stands out for assessing the risk of positioning injuries, identifying the incidence of skin injuries and considering neuropathic injuries, offering suggestions for coping strategies.

### Study limitations

It should be noted that not all patients were assessed at the time of hospital discharge. Furthermore, since these were elective surgeries with short hospital stays, it is unknown whether skin lesions may have appeared at home and were not recorded.

### Contributions to nursing

This study makes a significant contribution to perioperative nursing, as it not only assesses the risk of developing surgical positioning injuries in a medium-sized hospital performing elective surgeries, but also identifies the incidence of skin lesions and peripheral neuropathy. In this way, perioperative nursing can develop strategies and interventions for planning individualized care. This study also validates and acknowledges that the nursing team faces barriers, making the situation even more challenging for professionals.

## CONCLUSIONS

Performing risk assessment using a validated scale is a valuable resource for the perioperative nurse, ensuring quality of care and patient safety. This practice should be used more in institutions to systematize nursing care.

The findings showed an incidence of peripheral neuropathy after surgical procedures and a small incidence of skin lesions, highlighting the importance of training programs to assess the risk of these lesions. This includes the application of strategies such as the use of appropriate support surfaces, prophylactic materials, and the education of patients and staff.

The distinguishing feature of this study was, in addition to assessing the incidence of skin lesions, focusing on peripheral neuropathies. The importance of perioperative nursing conducting more studies in this area is highlighted, as research on the incidence of complications is essential to create risk reduction strategies based on situational diagnoses.

## Data Availability

The research data are available within the article.
