# Supplementary material for: Risk assessment for injuries resulting from positioning and incidence of neuropathy and skin lesions
Source: Rev Bras Enferm. 2026 Sep 28;79:e20250126. doi: 10.1590/0034-7167-2025-0126 (PMC13623297; doi:10.1590/0034-7167-2025-0126)
Supplement: Translation

# Translation: Avaliação do risco para lesões decorrentes do posicionamento e incidência de neuropatia e lesões tegumentares

**DOI:** 10.1590/0034-7167-2025-0126pt

**Authors:** Renata Elizabete Pagotti da Fonseca, Thamires Carraro Gatto, Carina Aparecida Marosti Dessotte

**Affiliations:** I Universidade de São Paulo. Ribeirão Preto, São Paulo, Brasil; II Universidade Federal de São Carlos. São Carlos, São Paulo, Brasil

**Keywords:** Úlcera por Pressão, Neuropatia de Pequenas Fibras, Posicionamento do Paciente, Enfermagem Perioperatória, Segurança do Paciente.

## Abstract

**Objetivos:**

avaliar o risco para desenvolver lesões decorrentes do posicionamento cirúrgico em pacientes adultos submetidos a cirurgias eletivas e identificar a incidência de complicações neurológicas e lesões de pele no perioperatório.

**Métodos:**

estudo observacional, longitudinal, prospectivo e quantitativo, desenvolvido em hospital de porte médio do interior paulista, Brasil.

**Resultados:**

a amostra do estudo era predominantemente feminina (57%), com mediana de 55 anos. Desses, 72,8% apresentavam doenças pré-existentes, com sobrepeso (41,8%) ou obesidade (34,8%). O escore da Escala de Avaliação de Risco para o Desenvolvimento de Lesões Decorrentes do Posicionamento Cirúrgico foi de baixo risco (75,9%), com incidência de 6,4% de neuropatia periférica e 0,8% de lesão por pressão (estágio 1/região sacral).

**Conclusões:**

utilizar escalas de avaliação de risco para desenvolvimento de lesões colabora para sistematizar assistência de enfermagem. Estudos sobre incidência de lesões (pele e neuropatia periférica) são essenciais para diagnóstico situacional e posterior criação de estratégias para reduzir riscos.

## INTRODUÇÃO

Na área da saúde, a qualidade e a segurança do paciente têm um significado especial devido às características do processo de trabalho^(1)^. No centro cirúrgico, é essencial focar nas complicações do período perioperatório para adotar estratégias que minimizem riscos e promovam excelência no cuidado.

Este estudo enfoca complicações decorrentes do posicionamento cirúrgico. Diversos fatores de risco, como idade, peso, condição da pele, estado nutricional, comorbidades (especialmente nos sistemas circulatório, respiratório e neurológico), imunossupressão e limitações físicas, devem ser investigados. Também é importante avaliar fatores intraoperatórios, como tipo de anestesia, tempo de cirurgia e posição cirúrgica^(2,3)^. Atenção especial deve ser dada a pacientes idosos, obesos e gestantes^(2,4)^.

Foram criadas e validadas escalas para avaliar o risco de lesões por posicionamento cirúrgico. Este estudo utilizou a Escala de Avaliação de Risco para o Desenvolvimento de Lesões Decorrentes do Posicionamento Cirúrgico (ELPO), desenvolvida no Brasil, que abrange sete itens: tipo de posicionamento na mesa cirúrgica; tempo de cirurgia; tipo de anestesia; superfície de suporte; posição dos membros; comorbidades; e idade do paciente^(5)^.

Apesar de essas complicações ocorrerem em vários sistemas, como circulatório, respiratório, osteoarticular, muscular, neurológico e tegumentar^(6)^, realizou-se a investigação das lesões tegumentares e neuropatias periféricas.

Os pacientes estão em alto risco de desenvolver lesões por pressão (LPs) durante procedimentos operatórios ou invasivos, devido à imobilidade prolongada no período perioperatório^(3)^. Forças físicas, como pressão, cisalhamento, microclima e fricção, utilizadas para manter uma posição cirúrgica adequada, podem lesionar a pele e tecidos subjacentes. Além disso, as condições da sala cirúrgica, como umidade e temperatura, aumentam a vulnerabilidade da pele^(3)^. Pacientes anestesiados podem não sentir dor ou desconforto durante os procedimentos^(2)^.

As LPs decorrentes do posicionamento cirúrgico são eventos adversos evitáveis, com incidência variando de 4% a 45%^(7)^. Essa variação pode ser atribuída à dificuldade de associar essas lesões ao posicionamento intraoperatório, especialmente devido à manifestação tardia, que pode ocorrer em até cinco dias, confundindo-se com outras causas não relacionadas ao ato cirúrgico^(8)^.

No período perioperatório, os pacientes também podem desenvolver neuropatia periférica iatrogênica, que é a lesão dos nervos responsáveis por transmitir mensagens para e do sistema nervoso central e da medula espinhal. As principais causas dessa lesão neurológica são o bloqueio de nervos periféricos e o posicionamento inadequado do paciente^(9)^. Entre 1990 e 2013, análise do banco de dados de reclamações da *American Society of Anesthesiologists* (ASA) mostrou que a incidência de lesão neurológica em pacientes sob anestesia geral foi de 12%^(10)^.

Os nervos periféricos podem ser lesionados durante o posicionamento, comprometendo funções sensoriais e motoras. Os principais mecanismos de lesão incluem compressão, isquemia e estiramento^(11)^. Fatores de risco incluem características do paciente e detalhes dos procedimentos anestésico e cirúrgico^(3,4)^. A neuropatia periférica perioperatória é avaliada por sinais e sintomas pós-operatórios, como parestesias, fraqueza muscular, formigamento ou dor nas extremidades^(12)^.

Essas complicações impactam custos governamentais e a qualidade de vida dos pacientes e suas famílias^(13)^. Portanto, é essencial avaliar o risco de lesões decorrentes do posicionamento cirúrgico e investigar complicações neurológicas e de pele em cirurgias eletivas. O diagnóstico situacional orienta profissionais na identificação de riscos, fundamentando decisões assertivas. Além disso, há uma lacuna em estudos sobre neuropatia periférica no cuidado de enfermagem perioperatório.

Nesse contexto, foram formuladas as seguintes questões de pesquisa: qual a classificação de risco para o desenvolvimento de lesões decorrentes do posicionamento cirúrgico de pacientes adultos submetidos a cirurgias eletivas em hospital universitário do interior paulista? Qual é a incidência de lesões neurológicas decorrentes do posicionamento cirúrgico (neuropatia periférica iatrogênica) e lesões de pele (LP) em pacientes adultos submetidos a cirurgias eletivas em hospital universitário do interior paulista?

## OBJETIVOS

Avaliar o risco para o desenvolvimento de lesões decorrente do posicionamento cirúrgico em pacientes adultos submetidos a cirurgias eletivas e identificar a incidência de lesões decorrentes do posicionamento cirúrgico, como neuropatia periférica iatrogênica e lesões de pele (LP), em pacientes adultos submetidos a cirurgias eletivas em hospital universitário no interior paulista.

## MÉTODOS

### Aspectos éticos

O presente estudo segue as normas brasileiras estabelecidas pelas Resoluções n^o^ 466/2012^(14)^ e n^o^ 510/2016^(15)^ do Conselho Nacional de Saúde, as quais regulamentam a pesquisa em seres humanos. O projeto foi submetido ao Comitê de Ética em Pesquisa e recebeu parecer favorável. A participação dos envolvidos na pesquisa ocorreu mediante a assinatura do Termo de Consentimento Livre e Esclarecido.

### Desenho, período e local do estudo

Trata-se de estudo observacional, longitudinal e prospectivo, desenvolvido em hospital universitário de média complexidade e médio porte do interior paulista, que seguiu os critérios estabelecidos de acordo com a ferramenta *STrengthening the Reporting of OBservational studies in Epidemiology* ^(16)^. O hospital possui cinco salas cirúrgicas e realiza uma média de 150 cirurgias por mês.

A coleta de dados ocorreu nos meses de julho a dezembro de 2024, e foi realizada da seguinte forma: os dados sociodemográficos e clínicos, bem como os dados do período pré-operatório, foram coletados no consultório do centro cirúrgico. No período intraoperatório, a coleta de dados ocorreu desde a entrada do paciente na sala operatória até sua transferência para a recuperação pós-anestésica. Durante esse período, foram coletados dados referentes ao procedimento anestésico-cirúrgico, incluindo a aplicação da ELPO. No período pós-operatório, para identificar as possíveis lesões neuropáticas e tegumentares, o paciente foi avaliado durante a transferência da mesa cirúrgica para a maca, no período pós-operatório imediato, na sala de recuperação pós-anestésica e diariamente até o dia da alta hospitalar.

### Amostra, critérios de inclusão e exclusão

Amostra consecutiva e não probabilística foi constituída por pacientes de ambos os sexos, maiores de 18 anos, que foram submetidos a procedimentos cirúrgicos eletivos nas especialidades de cirurgia geral, cabeça e pescoço, ginecologia, mastologia, ortopedia, oftalmologia, proctologia, urologia e cirurgia vascular. Para responder ao segundo objetivo proposto, foram excluídos pacientes com presença de neuropatia periférica e LPs prévias à entrada no centro cirúrgico.

### Protocolo do estudo

Para a coleta de dados sociodemográficos e clínicos, bem como dos períodos pré-, intra e pós-operatórios, foi utilizado um instrumento adaptado de Lopes *et al*. abrangendo as seguintes variáveis^(5)^: data de nascimento; data da cirurgia; nome da cirurgia; sexo biológico; peso; altura; classificação do estado físico conforme a ASA; e presença de doenças pré-existentes (diabetes mellitus, doença vascular prévia, neuropatia prévia, trombose venosa profunda, hipertensão arterial sistêmica (HAS), LP prévia). Todas as variáveis foram coletadas do prontuário do paciente. A idade foi calculada subtraindo a data da entrevista da data de nascimento. O Índice de Massa Corporal (IMC) foi calculado utilizando o peso e a altura coletados. Para a classificação nutricional em adultos, foi considerado o parâmetro recomendado pela Organização Mundial da Saúde de IMC^(17)^.

A variável “presença de neuropatia periférica” prévia foi coletada do prontuário do paciente, considerando a avaliação pré-operatória, e foi classificada como “presença de neuropatia periférica prévia: sim ou não”.

Para a obtenção da variável “LP prévia”, a pesquisadora realizou a avaliação do paciente no pré-operatório, por meio de inspeção de toda a superfície corporal, e foi classificada como “presença de LP prévia: sim ou não”.

Foram coletadas também as variáveis pré-operatórias: presença de dor (sim ou não); local; avaliação da dor utilizando uma escala numérica; presença de LP (sim ou não); e história prévia de LP (sim, não, local). No período intraoperatório, foram coletadas as variáveis “horário do início da anestesia e da cirurgia” e “horário do término da anestesia e da cirurgia”.

No período intraoperatório, foi aplicada a ELPO^(5)^, composta de sete itens: tipo de posição cirúrgica; tempo de cirurgia; tipo de anestesia; superfície de suporte; posição dos membros; comorbidades; e idade do paciente. Cada item apresenta cinco subitens, com pontuação entre 1 e 5. Ao final, obteve-se o escore da escala, que pode variar entre 7 e 35 pontos. Quanto maior o escore em que o paciente é classificado, maior o risco para desenvolver lesões decorrentes do posicionamento cirúrgico. O escore 20 é respeitado como ponto de corte para diferenciar os pacientes da seguinte forma: pontuação entre 7 e 19 pontos é considerada como menor risco; e pontuação entre 20 e 35 pontos é considerada maior risco para desenvolvimento de LP decorrente do posicionamento cirúrgico^(5)^.

No período pós-operatório, foram coletados os seguintes dados: presença de dor; parestesia; fraqueza muscular; e formigamento em local que não fosse o sítio cirúrgico. Considerando o conceito de neuropatia periférica adotado no estudo, o paciente foi classificado como “sim” para a presença de lesão nervosa - neuropatia periférica - se apresentasse pelo menos um desses sinais em qualquer momento da coleta de dados no período pós-operatório.

Além disso, a pele foi avaliada quanto à presença de marcas deixadas por lençóis ou acessórios de equipamentos, eritema branqueável e lesões de pele. Para identificar a LP, toda a extensão da pele do paciente foi avaliada por meio de inspeção e palpação em todos os períodos de avaliação. Para a classificação do estágio da LP, foi utilizado o critério da *National Pressure Injury Advisory Panel* ^(18,19)^. Para que o estudo seja replicável, o passo a passo foi reproduzido na Figura 1.

**Figura 1:** Linha do tempo da coleta de dados, 2025

## RESULTADOS

### Avaliação do risco de lesão decorrente do posicionamento cirúrgico

Para responder ao primeiro objetivo proposto, que é avaliar o risco para o desenvolvimento de lesões decorrentes do posicionamento cirúrgico, a amostra foi composta por 158 pacientes, sendo a maioria do sexo feminino (n=90, 57%). As idades variaram de 20 a 82 anos, com mediana de 55 anos. A maioria dos pacientes foi classificada como ASA II (n=111, 70,3%), seguida por ASA I (n=40, 25,3%) e ASA III (n=7, 4,4%). Na avaliação nutricional de acordo com o IMC dos pacientes entrevistados, a maioria dos pacientes entrevistados apresentava sobrepeso (n=66, 41,8%), seguida por obesidade (n=55, 34,8%) e, por fim, eutrofia (n=37, 23,4%).

Com relação às doenças pré-existentes, 72,8% da amostra (n=115) relatou ter alguma doença pré-existente antes da realização do procedimento cirúrgico. Houve prevalência de pacientes hipertensos (n=63, 39,9%) ou com outra doença vascular (n=48, 30,4%), seguidos por diabéticos (n=34, 21,5%) e portadores de neuropatia periférica (n=33, 20,9%). Além disso, uma pequena amostra (n=25, 15,8%) relatou ser tabagista.

Na variável “ocupação dos participantes da pesquisa”, a maior frequência foi de atividades realizadas no lar (n=30, 18,9%), seguidas por atividades auxiliares (n=23, 14,5%) e aposentados (n=20, 12,6%), conforme disposto na Tabela 1.

**Tabela 1:** Frequência das ocupações dos participantes de pesquisa, São Carlos, São Paulo, Brasil

Ocupação	Frequência
Aposentado	20 (12,6%)
Auxiliar de serviços (montagem, produção, limpeza)	23 (14,5%)
Auxiliar administrativo (secretária)	4 (2,5%)
Carpinteiro/jardineiro/serralheiro/marceneiro	8 (5,0%)
Comerciante	15 (9,5%)
Cozinheiro/padeiro/costureira	12 (7,6%)
Desempregado	1 (0,6%)
Do lar	30 (18,9%)
Estudante	4 (2,5%)
Motorista	5 (3,2%)
Pintor/eletricista/pedreiro	9 (5,7%)
Produtor agrícola	3 (2,0%)
Professor	3 (2,0%)
Profissional de saúde	12 (7,6%)
Segurança/vigilante/zelador	6 (3,8%)
Servidor público	3 (2,0%)
**TOTAL**	**158 (100%)**

No período de coleta de dados, a especialidade que mais realizou procedimentos foi a cirurgia geral, incluindo colecistectomias por videocirurgia, exéreses de lesões e hernioplastias (n=52, 32,9%), seguida por cirurgias ginecológicas, com ênfase em colpoperineoplastias, exéreses de endometriomas, histerectomias e histeroscopias (n=24, 15,2%). Outras especialidades incluíram cirurgia vascular (n=22, 13,9%), ortopedia (n=18, 11,4%), oftalmologia (n=17, 10,7%), urologia (n=12, 7,6%), proctologia (n=5, 3,2%) e mastologia (n=5, 3,2%). Cabe ressaltar, para entendimento do porte da instituição, que as cirurgias oftalmológicas foram exclusivas para pterígio; as ortopédicas, para túnel do carpo; as de cabeça e pescoço, para paratireoidectomia e tireoidectomia; as de mastologia, para quadrantectomia; e as vasculares, para tratamento cirúrgico de varizes.

A análise de risco desses participantes de pesquisa evidenciou que a maioria apresentou escore de baixo risco (n=120, 75,9%). A Figura 2 destaca o escore da escala de acordo com o número absoluto dos pacientes.

**Figura 2:** Pontuação dos participantes de pesquisa segundo a Escala de Avaliação de Risco para o Desenvolvimento de Lesões Decorrentes do Posicionamento Cirúrgico, 2025

A maior parte dos procedimentos foi realizada sob raquianestesia (n=75, 47,5%). A posição cirúrgica que mais prevaleceu foi a supina (n=102, 64,6%), e a maioria das cirurgias teve duração de até uma hora (n=79, 50%) ou, no máximo, duas horas (n=60, 38%), evidenciando que o hospital atende cirurgias de pequeno e médio porte. Como superfície de suporte, foi utilizado o próprio colchão da mesa operatória e coxins de algodão, e a maior parte dos membros superiores foi posicionada com abertura menor que 90º (n=132, 83,5%), conforme especificado na Tabela 2.

**Tabela 2:** Apresentação da avaliação de risco para desenvolvimento de lesão segundo a frequência na amostra estudada, São Carlos, São Paulo, Brasil

Itens avaliados na escala		Frequência
**Tipo de posição cirúrgica**	Supina	102 (64,6%)
	Lateral	6 (3,8%)
	Trendelenburg	17 (10,8%)
	Prona	8 (5,1%)
	Litotômica	25 (15,8%)
**Tempo de cirurgia**	Até uma hora	79 (50%)
	Acima de uma hora até duas horas	60 (38%)
	Acima de duas horas até quatro horas	18 (11,4%)
	Acima de quatro horas até seis horas	1 (0,6%)
	Acima de seis horas	0 (0%)
**Tipo de anestesia**	Local	37 (23,4%)
	Sedação	13 (8,2%)
	Regional	75 (47,5%)
	Geral	30 (19%)
	Geral e regional	3 (1,9%)
**Superfície de suporte**	Colchão da mesa cirúrgica (espuma convencional)	158 (100%)
**Posição dos membros**	Abertura dos membros inferiores menores que 90º	132 (83,5%)
	Elevação dos joelhos maior que 90º	26 (16,5)
**Comorbidades**	Sem comorbidades	38 (24,1%)
	Doença vascular	29 (18,4%)
	Diabetes mellitus	10 (6,3%)
	Desnutrição ou obesidade	48 (30,4%)
	Neuropatia periférica	33 (20,9%)
**Idade do paciente**	Entre 18 e 39 anos	36 (22,8%)
	Entre 40 e 59 anos	71 (44,9%)
	Entre 60 e 69 anos	37 (23,4%)
	Entre 70 e 79 anos	12 (7,6%)
	Maior que 80 anos	2 (1,3%)

Salienta-se a preocupação em iniciar procedimentos cirúrgicos mais longos nessa instituição sem a implantação de estratégias de prevenção dessas complicações, pois o aumento da complexidade e do tempo cirúrgico tornaria o risco ainda mais elevado nessa amostra, que já apresenta outras doenças associadas, além do sobrepeso e da obesidade.

### Incidência de complicações neurológicas e lesão de pele

Para identificar a incidência de complicações neurológicas e lesões de pele no período perioperatório, 33 pacientes da amostra inicial foram excluídos nessa fase, pois possuíam neuropatia periférica prévia. Foi importante considerá-los na primeira fase do estudo, pois pacientes com neuropatia periférica têm uma pontuação maior na escala de risco. Se fossem excluídos nessa primeira fase, o risco de desenvolvimento de lesões estaria enviesado.

Assim, na segunda fase, participaram 125 pacientes, sendo 65 do sexo feminino (52,4%). Para identificar a incidência de neuropatia periférica, foram excluídos os pacientes que já possuíam o diagnóstico e/ou que compareceram ao serviço de saúde com dor não relacionada ao sítio cirúrgico no dia da cirurgia. Nenhum participante de pesquisa apresentou fraqueza muscular ou formigamento nas extremidades, porém alguns pacientes apresentaram dor e parestesia após a intervenção. Assim, a incidência de neuropatia periférica neste estudo foi de 6,4% (n=8), sendo que sete pacientes apresentaram dor, e um paciente, parestesia. Desses pacientes, dois apresentaram dor no ombro direito, três no membro superior direito (MSD), um no ombro direito e no MSD, e um paciente apresentou dor na coxa esquerda. É importante salientar que o paciente que relatou dores na coxa esquerda havia realizado procedimento cirúrgico em posição litotômica.

Com relação às lesões tegumentares decorrentes do posicionamento cirúrgico, a inspeção da pele se deu com observação de três sinais: marcas de lençol ou acessórios de equipamentos na pele; eritema branqueável; e LP. Constatou-se que 73,6% dos pacientes, no momento da transferência da mesa cirúrgica para a maca, estavam com a pele marcada por lençóis ou acessórios de equipamentos (esfigmomanômetro, oxímetro de pulso, placa de bisturi) e 31,2% apresentavam eritema branqueável em alguma parte do corpo nesse mesmo momento da inspeção. No período pós-operatório imediato, 29,6% da amostra mantinha as marcas de lençol (n=37), e 10,4% ainda apresentava eritema branqueável (n=13). Apenas um paciente (0,8%) apresentou LP estágio 1 na região sacral. Esse paciente era um adulto de 51 anos, com sobrepeso, hipertenso, submetido à raquianestesia e a um procedimento urológico, e tinha escore ELPO de alto risco.

No tocante aos locais do aparecimento dessas marcas e eritemas, destacam-se a região dorsal, com marcas de lençóis e acessórios de equipamentos, e a região sacral, com presença de eritema branqueável logo após o término da cirurgia, no momento da transferência do paciente da mesa cirúrgica para a maca, conforme demonstra a Figura 3.

**Figura 3:** Áreas com presença de marcas de pele e eritema branqueável na amostra estudada, 2025

Nessa amostra estudada, o tempo de internação variou com alta no mesmo dia (n=71, 56,8%), com um dia de internação (n= 50, 40%), dois dias (n=2, 1,6%), três (n=1, 0,8%) e quatro dias (n=1, 0,8%). Cabe ressaltar que o paciente que apresentou a LP foi o que ficou mais dias internado (quatro dias).

## DISCUSSÃO

No presente estudo, a maioria dos participantes era do sexo feminino (57%) e apresentava comorbidades (72,8%), que são conhecidas por aumentar o risco de desenvolvimento de LPs decorrentes do posicionamento cirúrgico^(6)^. As doenças mais frequentes foram HAS (39,9%) e outras doenças vasculares (30,4%).

A HAS afeta a circulação sanguínea, comprometendo as capacidades sensoriais e de oxigenação de órgãos e tecidos, tornando-se um fator de risco para LP^(20,21)^. Pacientes com diabetes mellitus (21,5% da amostra) também têm maior risco de LP devido à vasculopatia, que diminui o fluxo sanguíneo e compromete a perfusão tecidual^(20,22)^.

Comorbidades como doenças vasculares, respiratórias, neuropatias e desnutrição aumentam a fragilidade dos sistemas do corpo. Quanto mais graves essas condições, especialmente as que afetam a perfusão e oxigenação tecidual, maior o risco de lesões por posicionamento cirúrgico^(6)^. Avaliar riscos de complicações perioperatórias é essencial para que pacientes, familiares e a equipe multiprofissional escolham o melhor cuidado durante o período perioperatório.

Um resultado preocupante foi a alta prevalência de sobrepeso (41,8%) e obesidade (34,8%). A obesidade é uma consideração importante no ambiente perioperatório, pois sua incidência está aumentando, e pacientes obesos têm reserva respiratória diminuída e risco aumentado de complicações no posicionamento cirúrgico^(3)^. A equipe de enfermagem perioperatória deve oferecer o melhor cuidado possível a essa população e adotar estratégias para garantir o melhor posicionamento. Estudo destacou a necessidade de avaliar a bioimpedância como fator de risco para o desenvolvimento de LP, considerando a distribuição da pressão em regiões propensas ao contato com superfícies de suporte^(23)^.

Outro fator de risco considerado foi a idade, com 32,3% da amostra composta por idosos. Pacientes idosos apresentam alterações fisiológicas, perda de elasticidade da pele e prejuízos na nutrição e oxigenação dos tecidos^(20,22-24)^, tornando-os mais suscetíveis a complicações do posicionamento cirúrgico.

Na avaliação do escore de risco, prevaleceu o baixo risco (75,9%). Apesar da presença de fatores de risco como sobrepeso, obesidade e comorbidades, as cirurgias eram, em geral, de curta duração: 50% delas duravam menos de uma hora, e 38%, até duas horas. Isso destaca a importância de programas de capacitação para avaliar riscos, aplicar condutas adequadas, transferir cuidados para outras unidades e notificar eventos adversos, garantindo a segurança do paciente^(2)^. Com o aumento da complexidade cirúrgica, o risco pode aumentar, ressaltando a necessidade de novas estratégias, uso de superfícies de suporte adequadas, materiais profiláticos^(2)^ e educação de pacientes e colaboradores enquanto o risco é baixo. Na era da inovação e novas tecnologias em saúde, a enfermagem deve estar equipada com novos conhecimentos para oferecer assistência de qualidade e garantir a segurança do paciente no período perioperatório.

O presente estudo apontou a posição supina como a mais prevalente, assim como outros estudos, que também a apontam como a posição mais utilizada na maioria dos procedimentos cirúrgicos, além de ser a posição de escolha para a indução da anestesia^(6,25,26)^.

O diferencial deste estudo foi, além de avaliar a incidência de lesões tegumentares, focar nas neuropatias periféricas. Há poucos estudos na enfermagem perioperatória que avaliam a incidência de neuropatias periféricas no período perioperatório. Estudo brasileiro na área de anestesiologia relatou casos de neuropatia periférica pós-cirurgia bariátrica^(27)^.

A incidência de neuropatia periférica neste estudo foi de 6,4%. Esse resultado destaca a necessidade de monitoramento contínuo e estratégias de manejo adequadas para pacientes com neuropatia periférica após cirurgias, visando reduzir o impacto na qualidade de vida e promover a reabilitação. A enfermagem perioperatória deve realizar mais estudos sobre o tema, especialmente porque lesões de nervos periféricos podem evoluir para dor neuropática (DN) persistente, grave e refratária ao tratamento, afetando a qualidade de vida dos pacientes^(28)^.

Revisão mostrou que a dor crônica pós-operatória é experimentada por 10% a 50% dos indivíduos após operações clássicas, podendo ser grave em cerca de 5% a 10% desses pacientes, e 20% procuram uma clínica de dor por causa de dor pós-operatória crônica. A incidência de DN varia entre 6% e 68%, dependendo do tipo de cirurgia^(29)^.

Estudo aborda a neuropatia como complicação após artroplastia de quadril, afetando até 3% dos pacientes e resultando em DN e fraqueza muscular. O estudo enfatiza a importância de uma reabilitação precoce e multidisciplinar para minimizar danos neuropáticos relacionados à manipulação cirúrgica^(30)^.

A incidência de LPs foi de 0,8% (lesão em estágio 1 na região sacral). No entanto, 73,6% dos pacientes apresentaram marcas na pele de lençóis ou acessórios durante a transferência da mesa cirúrgica para a maca, e 31,2% tinham eritema em alguma parte do corpo nesse momento. Estudo similar encontrou a mesma taxa de LP na região sacral^(24)^.

Para evitar marcas na pele, enfermeiros perioperatórios devem garantir que a superfície da cama esteja lisa e sem rugas antes de posicionar os pacientes^(2)^. Estudo de 1989 já indicava que a pressão sacral aumenta com camadas adicionais, como lençóis dobrados e almofadas para incontinência, em pacientes na posição supina^(31)^.

O correto posicionamento é essencial para a cirurgia, garantindo boa exposição do sítio cirúrgico, mas deve manter a anatomia corporal e ser fisiologicamente tolerado. Isso envolve a preferência do cirurgião, a necessidade do anestesiologista e as condições pré-existentes do paciente. A combinação desses fatores pode aumentar o risco de complicações pós-operatórias, sejam tegumentares ou neuropáticas^(4-6)^.

Estudo brasileiro encontrou LP em 5,47% dos 128 pacientes acompanhados nos períodos intra e pós-operatórios em hospital de grande porte^(32)^. Outro estudo, com 52 pacientes, constatou que 7,7% desenvolveram LPs decorrentes do procedimento cirúrgico. Neste estudo, a maioria dos pacientes (65,4%) também tinha baixo risco de lesão por posicionamento e utilizava um colchão convencional de espuma, o que é similar ao presente estudo^(25)^.

Estudos sobre a incidência de complicações são essenciais, pois seus resultados podem orientar estratégias para reduzir riscos. Pesquisa com 944 pacientes de alto risco para lesões de posicionamento mostrou redução na incidência dessas lesões (de 4,8% para 1,6%) após a implementação de medidas preventivas, como educação dos pacientes, aplicação de coberturas protetoras, controle da umidade da pele e uso de superfícies de suporte^(8,33)^.

A equipe de enfermagem do centro cirúrgico deve rotineiramente avaliar pacientes em busca de LPs após a cirurgia e garantir o acompanhamento adequado, incluindo a transferência do cuidado sobre esses sinais. Devem avaliar o sistema tegumentar quanto a eritema que desaparece ao pressionar, mudanças na temperatura da pele, textura ou sensação de dor^(34)^, além de investigar dor, parestesias, fraqueza muscular e formigamento em áreas fora do sítio cirúrgico.

Entretanto, a equipe de enfermagem enfrenta desafios na promoção de atendimento humanizado no centro cirúrgico. Revisão sistemática destacou desafios como sobrecarga de trabalho, pressão por eficiência, impacto da tecnologia na humanização, educação e capacitação em humanização e posicionamento correto do paciente, além de barreiras culturais e organizacionais^(8)^. A enfermagem perioperatória deve estar preparada para superar esses desafios, sempre visando à segurança do paciente como prioridade.

A equipe de enfermagem já possui diretrizes para conduzir o risco de lesões por posicionamento cirúrgico^(2,4,12,19,34)^, com orientações para prevenir complicações em pacientes submetidos a procedimentos operatórios e invasivos. É essencial fortalecer os serviços de saúde e capacitar profissionais perioperatórios para implementar programas de prevenção de complicações, realizar avaliações de risco pré-operatórias^(20)^, utilizar superfícies de suporte^(8)^ e materiais profiláticos, manter comunicação eficaz durante a transferência de cuidados e estabelecer políticas e procedimentos^(22)^ que assegurem a qualidade da assistência e a educação de pacientes e colaboradores.

No entanto, ainda há desafios na busca pela segurança do paciente. Algumas instituições carecem de dispositivos especializados para alívio da pressão no período intraoperatório, recorrendo a adaptações com materiais como espuma, algodão e lençóis para reduzir a pressão de interface tecidual^(25)^.

Este trabalho se destaca por avaliar o risco de lesões por posicionamento, identificar a incidência de lesões tegumentares e considerar lesões neuropáticas, oferecendo sugestões de estratégias de enfrentamento.

### Limitações do estudo

Destaca-se que nem todos os pacientes foram avaliados no momento da alta hospitalar. Além disso, como eram cirurgias eletivas, com tempo de internação reduzido, não se sabe se lesões de pele podem ter aparecido em domicílio e não foram contabilizadas.

### Contribuições para a área da enfermagem

Este estudo contribui significativamente para a enfermagem perioperatória, pois não só avalia o risco de desenvolvimento de lesão por posicionamento cirúrgico em hospital de médio porte com cirurgias eletivas, como também identifica a incidência de lesões de pele e de neuropatia periférica. Dessa forma, a enfermagem perioperatória pode buscar estratégias e intervenções para o planejamento de um cuidado singular. Este estudo também valida e compreende que a equipe de enfermagem encontra barreiras, tornando-se, assim, ainda mais desafiador para o profissional.

## CONCLUSÕES

Realizar a avaliação de risco por meio de escala validada é um recurso valioso para o enfermeiro perioperatório, garantindo qualidade no atendimento e segurança ao paciente. Essa prática deve ser mais utilizada nas instituições para sistematizar o cuidado de enfermagem.

Os achados mostraram uma incidência de neuropatia periférica após procedimentos cirúrgicos e uma pequena incidência de lesões tegumentares, destacando a importância de programas de capacitação para avaliar o risco dessas lesões. Isso inclui a aplicação de estratégias, como o uso de superfícies de suporte adequadas, materiais profiláticos e a educação de pacientes e colaboradores.

O diferencial deste estudo foi, além de avaliar a incidência de lesões tegumentares, focar nas neuropatias periféricas. Destaca-se a importância de a enfermagem perioperatória realizar mais estudos nessa área, pois pesquisas sobre a incidência de complicações são essenciais para criar estratégias de redução de risco com base em diagnósticos situacionais.

## Data Availability

Os dados de pesquisa estão disponíveis no corpo do artigo.
